# Prevalence and determinants of depression among old age: a systematic review and meta-analysis

**DOI:** 10.1186/s12991-021-00375-x

**Published:** 2021-12-18

**Authors:** Yosef Zenebe, Baye Akele, Mulugeta W/Selassie, Mogesie Necho

**Affiliations:** 1 Department of Psychiatry, College of Medicine and Health Sciences, Wollo University, Dessie, Ethiopia; 2 Department of Pharmacy, College of Medicine and Health Sciences, Wollo University, Dessie, Ethiopia; 3 Department of Pediatrics and Child Health Nursing, College of Medicine and Health Sciences, Wollo University, Dessie, Ethiopia

**Keywords:** Depression, Elderly, Global

## Abstract

**Background:**

Depression is a leading cause of disability worldwide and is a major contributor to the overall global burden of disease. It is also one of the most common geriatric psychiatric disorders and a major risk factor for disability and mortality in elderly patients. Even though depression is a common mental health problem in the elderly population, it is undiagnosed in half of the cases. Several studies showed different and inconsistent prevalence rates in the world. Hence, this study aimed to fill the above gap by producing an average prevalence of depression and associated factors in old age.

**Objective:**

This study aims to conduct a systematic review and meta-analysis to provide a precise estimate of the prevalence of depression and its determinants among old age.

**Method:**

A comprehensive search of PubMed, Scopus, Web of sciences, Google Scholar, and Psych-info from database inception to January 2020. Moreover, the reference list of selected articles was looked at manually to have further eligible articles. The random-effects model was employed during the analysis. Stata-11 was used to determine the average prevalence of depression among old age. A sub-group analysis and sensitivity analysis were also run. A graphical inspection of the funnel plots and Egger’s publication bias plot test were checked for the occurrence of publication bias.

**Result:**

A search of the electronic and manual system resulted in 1263 articles. Nevertheless, after the huge screening, 42 relevant studies were identified, including, for this meta-analysis, *n* = 57,486 elderly populations. The average expected prevalence of depression among old age was 31.74% (95% CI 27.90, 35.59). In the sub-group analysis, the pooled prevalence was higher among developing countries; 40.78% than developed countries; 17.05%), studies utilized Geriatrics Depression Scale-30(GDS-30); 40.60% than studies that used GMS; 18.85%, study instrument, and studies having a lower sample size (40.12%) than studies with the higher sample; 20.19%.

**Conclusion:**

A high prevalence rate of depression among the old population in the world was unraveled. This study can be considered as an early warning and advised health professionals, health policymakers, and other pertinent stakeholders to take effective control measures and periodic care for the elderly population.

## Background

The elderly people are matured and experienced persons of any community. Their experience, wisdom, and foresight can be useful for development and progress; they are a valuable asset for any nation [1]. Despite their invaluable wisdom and insight, the aging of the world's population is causing extensive economic and social consequences globally [2]. The aging population has increased rapidly over the last decades owing to two significant factors, namely, the reduction in mortality and fertility rates and improved quality of life, leading to an increase in life expectancy worldwide [3–5]. Globally, the number and proportion of people aged 60 years and older in the population are increasing. In 2019, the number of people aged 60 years and older was 1 billion. This number will increase to 1.4 billion by 2030 and 2.1 billion by 2050. By 2050, 80% of all older people will live in low- and middle-income countries [6–8].

A high geriatric population leads to high geriatric psychiatric problems [9]. The elderly, in general, face various challenges that are associated with physical and psychological changes commonly associated with the aging process [10]. The incidence of mental health problems is expected to increase among adults in general as well as in older populations in particular [11].

Depression is a leading cause of disability worldwide and is a major contributor to the overall global burden of disease [12]. It is also one of the most common geriatric psychiatric disorders [13] and a major risk factor for disability and mortality in older patients [14]. Even though depression is a common mental health problem in the elderly population, it is undiagnosed in about 50% of cases. The estimates for the prevalence of depression in the aging differ greatly [15–17]. WHO estimated that the global depressive disorder among older adults ranged between 10 and 20% [18–21]. Among all mentally ill individuals, 40% were diagnosed to have a depressive disorder [22]. People with depressive disorder have a 40% greater chance of premature death than their counterparts [20].

Most of the time, the clinical picture of depression in old age is masked by memory difficulties with distress and anxiety symptoms; however, these problems are secondary to depression [23–25]. Numerous community-based studies showed that older adults experienced depression-related complications [26–30]. Depression amplifies the functional disabilities caused by physical illness, interferes with treatment and rehabilitation, and further contributes to a decline in the physical functioning of a person [31, 32]. It also has an economic impact on older adults due to its significant contribution to the rise of direct annual livelihood costs [33]. Hence, improvement of mental health among people in late life is considered to be medically urgent to prevent an increase in suicides in a progressively aging society.

Although real causes of depression remain not clear, psychological, social, and biological processes are thought to determine the etiology of depression and comorbid psychiatric diagnoses (e.g., anxiety and various personality disorders) [34]. Social scientists, postulating the psychosocial theory, posited that depression could be caused by a lack of interpersonal and communication skills, social support, and coping mechanisms [35]. Old biological theories stated depression is caused by a lack of monoamines in the brain. However, recent theories underscore the role of Brain-derived neurotrophic factor (BDNF) in the pathogenesis of depression [36]. In general, depression in the elderly is the result of a complex interaction of social, psychological, and biological factors [37, 38].

Different factors associated with geriatric depression, such as female sex [39–47], increasing age [37, 40, 41, 44, 46–49], being single or divorced [42], religion [50], lower educational attainment [39–42, 44], unemployment [38, 42], low income [37, 39, 40, 42, 44, 46, 51, 52], low self-esteem [53], childhood traumatic experiences [54], loneliness or living alone [40, 50, 51, 55], social deprivation [45, 46, 56], bereavement [39, 43, 57, 58], presence of chronic illness or poor health status [37, 39, 43–46, 49, 50, 56, 59–64], lack of health insurance [42], smoking habit [48], cognitive impairment [39, 43–47, 61] and a history of depression [43, 44, 47].

Compared with other health services, evidence of depressive disorders tends to be relatively poor. Therefore, the level of its burden among older adults is not well addressed in the world. Lack of adequate evidence about depression in older adults may be a factor that contributes to poor or inconsistent mental health care at the community level [21, 65]. In addition to the poor setting for mental health care services, there are no up-to-date systematic reviews and meta-analysis studies conducted that could vividly show the global prevalence and determinants of depression among old age. Several studies also revealed different and inconsistent prevalence rates in the world. Therefore, this systematic review and meta-analysis aimed to summarize the existing evidence on the prevalence of depression among old age and to formulate possible suggestions for clinicians, the research community, and policymakers.

## Methods

### Search process

A systematic search of the literature in September 2020 using both international [PubMed, Scopus, Web of sciences, Google Scholar, Psych-info, and national scientific databases] was conducted to identify English language studies, published between August 1994 and January 2020, that examined the prevalence of depression among old age. We searched English keywords of “epidemiology” OR “prevalence” OR “magnitude” OR “incidence” AND “factor” OR “associated factor” OR “risk” OR “risk factor” OR “determinant”, “depression”, “depressive disorder” OR “major depressive disorder” AND “old age” OR “elderly” OR “geriatrics”, “community”, “hospital” and “global”. In addition, the reference lists of the studies were manually checked to obtain further studies.

### Inclusion and exclusion criteria

Original quantitative studies that examined the prevalence and determinants of depression among old age were included. The included studies were randomized controlled trials, cohort, case–control, cross-sectional, articles written in English, full-text articles, and published between August 1994 and January 2020. The exclusion criteria were studies which published as review articles, qualitative studies, brief reports, letter to the editor or editorial comments, working papers articles published in a language other than English, researches conducted in non-human subjects, and studies having duplicate data with other studies. The literature search was conducted based on the PRISMA (preferred reporting items for systematic reviews and meta-analyses) guideline [66]. All articles were independently reviewed by four researchers against inclusion and exclusion criteria. Any initial disagreement was resolved.

### Data extraction and appraisal of study quality

After eliminating the duplicates, four investigators reviewed study titles and abstracts for eligibility. If at least one of them considered an article as potentially eligible, the full texts were assessed by the same reviewers. Any disagreements were resolved by discussion. Detailed information on the country, data source, study population, and results were extracted from each included study into a standardized spreadsheet by two authors and checked by the other two authors. EndNote X7.3.1 was used to organize the identified articles. Two investigators independently assessed the risk of bias of each of the included studies. The quality of studies included in the final analysis was evaluated with the Newcastle Ottawa quality assessment checklist [67]. The components of the quality assessment checklist include study participants and setting, research design, recruitment strategy, response rate, representativeness of the sample, the convention of valid measurement, reliability of measurement, and appropriate statistical analyses.

### Statistical analysis

The data were analyzed with STATA 12.0 [68]. Prevalence standard errors were calculated using the standard formula for proportions: sqrt [p*(1 – *p*)/*n*]; The heterogeneity across the studies in proportion of depression in the elderly population and the contribution of studies attributing to total heterogeneity was estimated by the I^2^ statistic. The point estimates from each study were combined using a random-effects meta-analysis model to obtain the overall estimate with the DerSimonian–Laird method. Sources of heterogeneity across studies were examined with meta-regression. Publication bias and small study effects were assessed with the Egger test.

## Results

### Search result

**Fig. 1:**
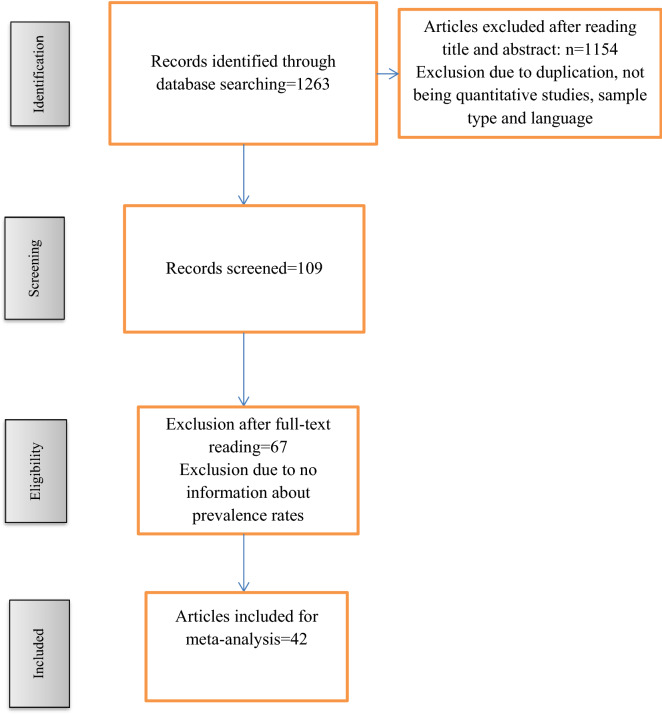
Articles search flow diagram

The search procedure primarily obtained *n* = 1263 results, which after reading the title and abstract, full-text, and the application of the inclusion and exclusion criteria were reduced to *n* = 42. The selection process is shown in Fig. 1.

### Characteristics of the study subjects

A total of 42 studies [38, 42, 50, 57, 69–105] studied our outcome of interest; A total sample size of fifty-seven thousand four hundred and eighty-six (57,486) elderly populations were included in the present study. The geographical province of studies was assessed. We found: Six studies in India [72, 86, 94, 95, 98, 102], five studies in China [50, 77, 84, 89], three studies in Turkey [71, 82, 105], three studies in Nepal [76, 90, 97], three studies in Thailand [70, 75, 83], two studies in the USA [91, 100], two studies in Australia [57, 99], two studies in Malaysia [42, 96], two studies in Ethiopia [81, 93], one study in German [103], one study in the UK [104], one study in Norway [85], one study in Italy [79], one study in Japan [87], one study in Mexico [78], one study in Brazil [92], one study in Finland [74], one study in Singapore [101], one study in Saudi Arabia [69], one study in the United Arab Emirates [80], one study in Ghana [88], one study in Sudan [73] and one study in Egypt [38]. Most of the studies in the present analysis were cross-sectional [38, 42, 50, 57, 69–79, 81, 82, 84–90, 92, 93, 95–98, 101–103, 105] and four studies were Cohort [85, 94, 99, 104].

Sixteen studies [70, 73, 74, 81, 86, 88, 90, 92–94, 97, 98, 102–105] used Geriatric Depression Scale-15 (GDS-15), 12 studies [38, 69, 71, 72, 75–77, 82, 84, 89, 96] used Geriatric Depression Scale-30 (GDS-30), four studies [50, 80, 83, 101] used Geriatric Mental State Schedule (GMS) and ten studies [42, 57, 78, 79, 85, 87, 91, 95, 99, 100] used others (ICD-10, CIDI, DASS-21, KICA, CES-D, Euro-D, DSM-III, MCS and HADS) tools to measure depression in old age (Table 1).

**Table 1:** Characteristics of study participants among the elderly populations

Author, year of publication	Country	Study design	Sample size	Tools with cut off points	Sampling technique	Response rate	Characteristics of respondents	Overall prevalence (%)
Boman et al. 2015	Anland, Finnish	CS	1452	GDS-15 ≥ 5	NR	93.5%	F ≥ 65 years	11.2
Güzel et al. 2020	Burdur, Turkey	CS	770	GDS-30 ≥ 14	Cluster sampling method	NR	M & F ≥ 65 years	51.8
Swarnalatha N et al. 2013	Chittoor District, India	CS	400	GDS-15 > 5	Random sampling	100%	M & F ≥ 60 years	47
Ashe et al. 2019	Cuttack district, India	CS	354	GDS-30 ≥ 10	Simple random sampling	97.5%	M & F > 60 years	81.1
Girma et al. 2016	Harar, Ethiopia	CS	344	GDS-15 ≥ 5	Systematic random sampling technique	97.7%	M & F > 60 years	28.5
Mirkena et al. 2018	Ambo, Ethiopia	CS	800	GDS-15 ≥ 5	Multi-stage sampling technique	94.8%	M & F ≥ 60 years	41.8
He et al. 2016	Rural China	CS	509	GDS-30 ≥ 11	NR	96.8%	M & F > 65 years	36.94
Cong et al. 2015	Fuzhou, China	CS	1910	GDS-30 ≥ 11	Randomly selected	98.0%	M & F > 60 years	10.5
Feng et al. 2014	Xinjiang, China	CS	1329	GMS ≥ 3	Multistage stratified random sampling	91.3%	M & F > 60 years	10.61
Kugbey et al. 2018	Ghana	CS	262	GDS-15 ≥ 5	Stratified random sampling	100%	M & F > 65 years	37.8
Rajkumar et al. 2009	Southern Indian, Tamil Nadu	CS	978	ICD-10	NR	97.75%	M & F > 65 years	12.7
Choulagai P S et al. 2013	Kathmandu Valley, Nepal	CS	78	GDS-30 ≥ 10	Purposively selected	100%	M & F > 60 years	51.3
Simkhada et al. 2017	Kathmandu, Nepal	CS	300	GDS-15 ≥ 5	Randomly selected	99.0%	M & F > 60 years	60.6
Manandhar et al. 2019	Kavre district, Nepal	CS	439	GDS-15 ≥ 6	Randomly selected	95.4%	M & F ≥ 60 years	53.1
Arslantas et al. 2014	Middle Anatolia, Turkey	CS	203	GDS-30 ≥ 13	NR	80.8%	M & F ≥ 65 years	45.8
Yaka et al. 2014	Turkey	CS	482	GDS-15 ≥ 8	Cluster sampling method	100%	M & F ≥ 65 years	18.5
Charoensakulchai et al. 2019	Thailand	CS	416	GDS-30 ≥ 13	NR	100%	M & F > 60 years	18.5
Forlani et al. 2012	Bologna, Italy	CS	359	ICD-10	Randomly chosen sample	100%	M & F > 74 years	25.1
Wilson et al. 2007	UK	Cohort	376	GDS-15 ≥ 5	NR	100%	M & F 80 to 90 years	21
Steffens et al. 2009	USA	Cohort	775	CIDI-SF ≥ 5	Stratified sampling method	90.5%	M & F > 71 years	11.19
Manaf et al. 2016	Perak, Malaysia	CS	230	DASS-21 ≥ 5	Convenient sampling	100%	M & F > 60 years	27.8
Almeida et al. 2014	Kimberley and Derby, Australia	CS	235	KICA-dep ≥ 9	NR	94.0%	M & F > 45 years	7.7
Weyerer et al. 2008	German	CS	3242	GDS-15 ≥ 6	NR	100%	M & F > 75 years	9.7
Jadav et al. 2017	Vadodara, Gujarat, India	CS	176	GDS-15 > 5	Simple random sampling	88%	M & F > 60 years	34.1
Sinha et al. 2013	Tamil Nadu, India	CS	103	GDS-15 ≥ 5	Universal sampling technique	100%	M & F ≥ 60 years	42.7
Kaji et al. 2010	Japan	CS	10,969	CES-D ≥ 16	Stratified sampling design	100%	M & F > 50 years	31.2
Ferna´ndez et al. 2014	Mexico	CS	7867	CES-D ≥ 5	NR	NR	M & F > 60 years	35.6
AL-shammari et al. 1999	Saudi Arabia	CS	7970	GDS-30 ≥ 10	Stratified two-stage sampling technique	98.8%	M & F > 60 years	39
Sidik et al. 2004	Sepang, Malaysia	CS	223	GDS-30 > 10	Simple random sampling	84.8%	M & F > 60 years	7.6
Subramaniam et al. 2016	Singapore	CS	2565	GMS ≥ 1	Stratified sampling design	NR	M & F > 60 years	17.1
Assil et al. 2013	Sudan	CS	300	GDS-15 ≥ 5	Systematic random sampling	100%	M & F > 60 years	41.0
Haseen et al. 2011	Rural, Thailand	CS	1001	Euro-D scale-12 ≥ 5	NR	100%	M & F > 60 years	27.5
Ghubash et al. 2004	United Arab Emirates	CS	610	GMS-A3 ≥ 3	Selected by randomly	90.3%	M & F > 60 years	20.2
Abdo et al. 2011	Zagazig District, Egypt	CS	290	GDS-30 ≥ 10	Multistage random sampling technique	100%	M & F > 60 years	46. 6
Snowdon et al. 1994	Sydney, Australia	Cohort	146	DSM-III	Random sample	69%	M & F > 65 years	12.5
McCall et al. 2002	USA	CS	617	MCS ≥ 42	Simple random sampling	61.7%	M & F > 65 years	25
Li et al. 2016	China, CDEP	CS	4901	GDS-30 ≥ 11	Consecutively selected	NR	M & F > 60 years	11.6
Mendes et al. 2008	Brazil, Inpatients	CS	189	GDS-15 > 6	Randomly selected	100%	M & F > 60 years	56.1
Li et al. 2016	China, EMI	CS	2373	GDS-30 ≥ 11	Consecutively selected	NR	M & F > 60 years	18.1
Prashanth et al. 2015	India, Outpatient	Cohort	51	GDS-15 ≥ 5	NR	100%	M & F > 60 years	58.8
Helvik et al. 2010	Norway, Medical inpatients	CS	484	HADS ≥ 8	NR	100%	M & F > 65 years	10.3
Anantapong et al. 2017	Thailand, Outpatients	CS	408	GDS-15 > 5	Convenience sampling	100%	65–99 years	9.6

CDEP: community-dwelling elderly people; CES-D: Center for Epidemiologic Studies Depression Scale; CIDI-SF: Composite International Diagnostic Interview Short Form; CS: cross-sectional; DASS-21: Depression, Anxiety, and Stress Scale; DSM-III: diagnostic and Statistical Manual of Mental Disorders; EMI: elderly medical inpatients; GDS: Geriatric Depression Scale; GMS: Geriatric Mental State Schedule; HADS: Hospital Anxiety and Depression Scale; KICA-dep: Kimberley Indigenous Cognitive Assessment of Depression; MCS: mental component summary; NR: not reported; UK: United Kingdom; USA: United States of America

### Quality of included studies

The quality of 42 studies [38, 42, 50, 57, 69–105] was assessed with the modified Newcastle Ottawa quality assessment scale. This scale divides the total quality score into 3 ranges; a score of 7 to 10 as very good/good, a score of 5 to 6 as having satisfactory quality, and a quality score less than 5 as unsatisfactory. The majority (28 from the 42 studies) had scored good quality, nine had a satisfactory quality, and four of the studies had unsatisfactory quality**.**

### The prevalence of depression among old age

**Fig. 2:**
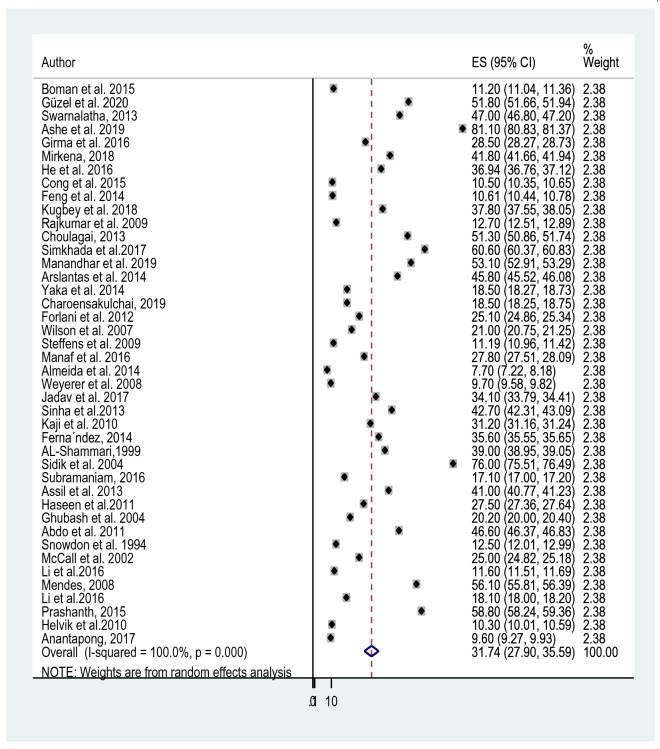
Forest plot for the prevalence of depression

The reported prevalence of elderly depression among 42 studies [38, 42, 50, 57, 69–105] included in this study ranges from 7.7% in a study from Malaysia and Australia [57, 96] to 81.1% in India [72]. The average prevalence of depression among old age using the random effect model was found to be 31.74% (95% CI 27.90, 35.59). This average prevalence of depression was with the heterogeneity of (*I*^2^ = 100%, *p* value = 0.000) from the difference between the 42 studies (Fig. 2).

### Subgroup analysis of the prevalence of depression among old age

**Fig. 3:**
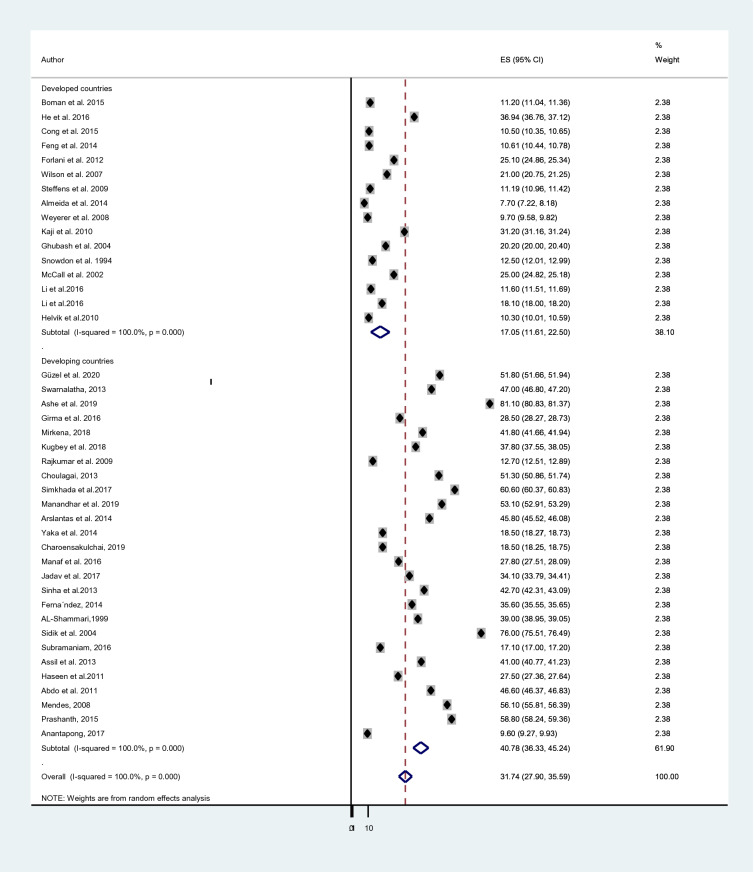
Sub-group analysis of depression based on economic status of countries

**Fig. 4:**
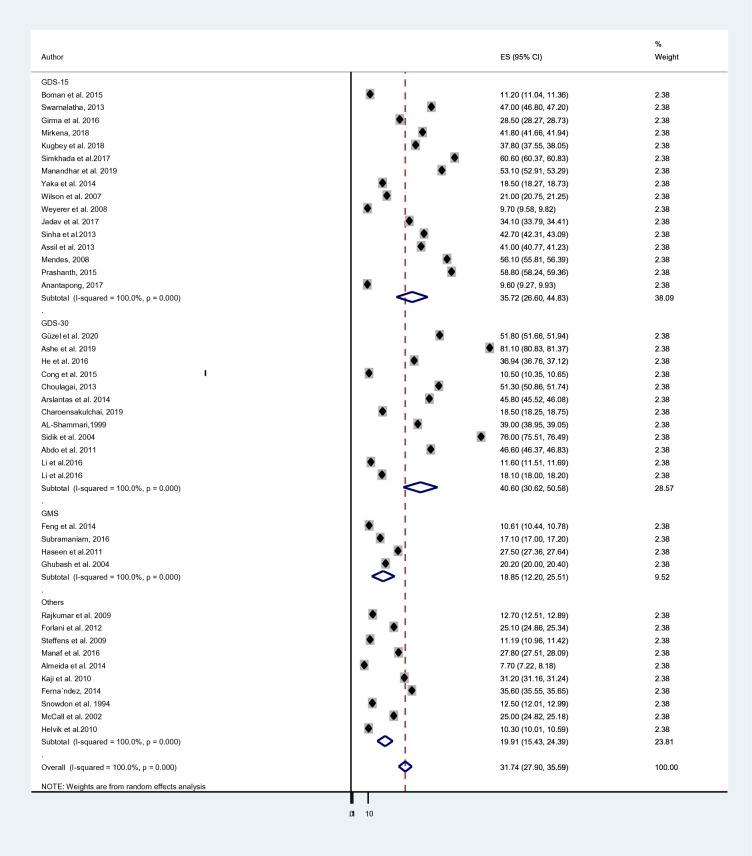
Sub-group analysis of depression based on study instruments

**Fig. 5:**
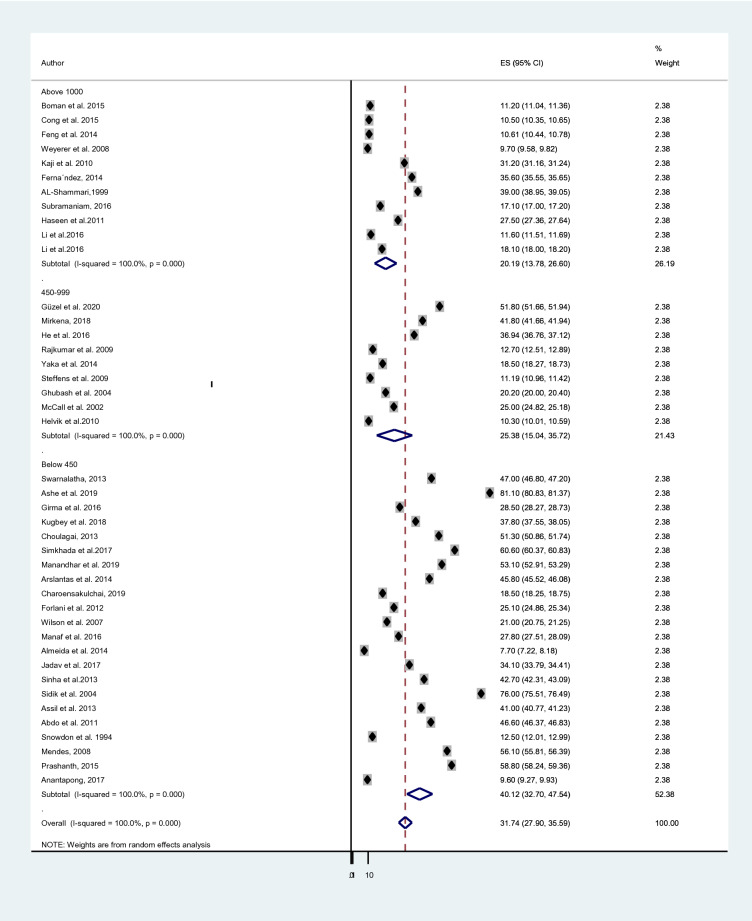
Sub-group analysis of depression based on sample size of studies

A subgroup analysis was done considering the economic status of countries, the study instrument and the sample size of each study. The cumulative prevalence of depression in elderly population among developing countries; 40.78% [38, 42, 69–73, 75, 76, 78, 81–83, 86, 88, 90, 92–98, 101, 102, 105] was higher than the prevalence in developed countries; 17.05% [50, 57, 74, 77, 79, 80, 84, 85, 87, 89, 91, 99, 100, 103, 104] (Fig. 3). The average prevalence of depression was 40.60% in studies that used GDS-30 [38, 69, 71, 72, 75–77, 82, 84, 89, 96] which is higher than the prevalence in studies that utilized GDS-15;35.72% [70, 73, 74, 81, 86, 88, 90, 92–94, 97, 98, 102–105], GMS;18.85% [50, 80, 83, 101] and other tools;19.91% [42, 57, 78, 79, 85, 87, 91, 95, 99, 100] (Fig. 4). Moreover, studies which had a sample size of below 450 [38, 42, 57, 70–73, 75, 76, 79, 81, 86, 88, 90, 92, 94, 96–99, 102, 104] provided higher prevalence of depression; 40.12% than those who had a sample size ranges from 450 to 999 [74, 80, 82, 84, 85, 91, 93, 95, 100, 105]; 25.38% and above 1000 [50, 69, 74, 77, 78, 83, 87, 89, 101, 103]; 20.19% (Fig. 5).

### Sensitivity analysis

**Fig. 6:**
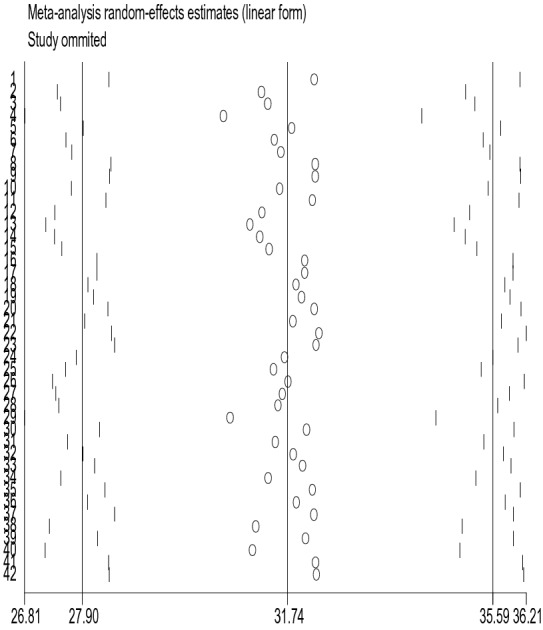
Sensitivity analysis for the prevalence of depression among old age

The sensitivity analysis was performed to identify whether one or more of the 42 studies had out-weighted the average prevalence of depression among old age. However, the result showed that there was no single influential study, since the 95% CI interval result was obtained when each of the 42 studies was excluded at a time (Fig. 6).

### Publication bias

**Fig. 7:**
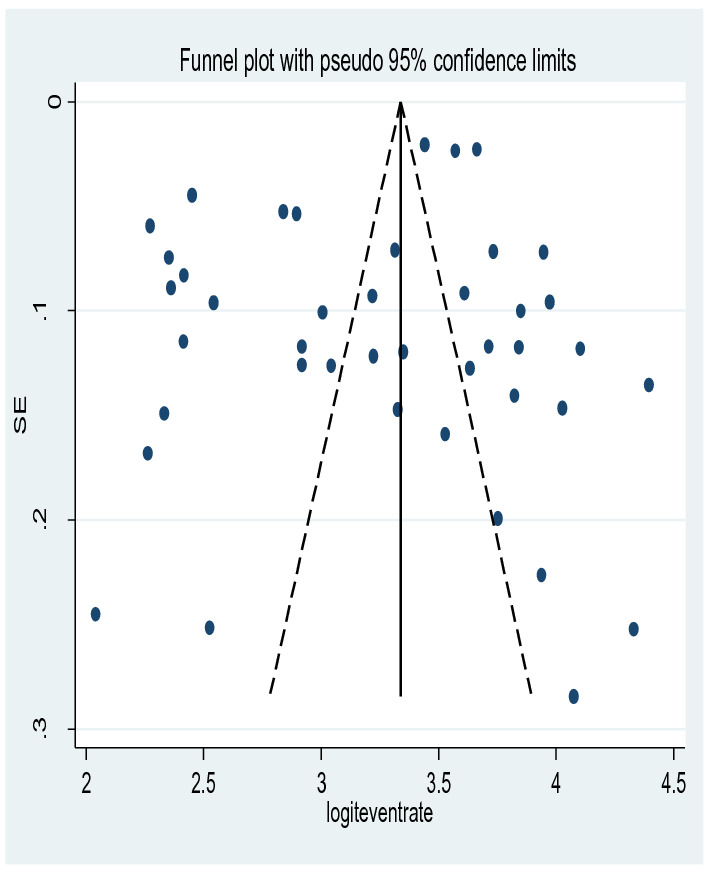
Funnel plot for publication bias for depression

There was no significant publication bias detected and Egger's test *p* value was (*p* = 0.644) showing the absence of publication bias for the prevalence of depression among old age. This was also supported by asymmetrical distribution on the funnel plot for a Logit event rate of prevalence of depression among old age against its standard error (Fig. 7).

### Factors associated with depression among old age

Among 42 studies [38, 42, 50, 57, 69–105] included in the present meta-analysis, only 32 [38, 42, 50, 57, 69, 72, 73, 75, 77–81, 83, 84, 86–98, 101–105] reported about the associated factors for depression among old age. Our qualitative synthesis for the sociodemographic factors associated with depression in elderly populations showed that female gender [38, 69, 72, 75, 80, 86, 89, 93, 98, 102, 105], age older than 75 years [38, 69, 101, 102], being single, divorced or widowed [38, 42, 69, 80, 81, 87, 89, 98, 105], being unemployed [69, 86, 96, 105], retired [95], no educational background [75, 81, 86, 89, 90, 97, 102] OR low level of education [69, 81, 84, 91, 92, 105], low level of income [69, 72, 78, 80, 94, 95, 105], substance use [75, 81, 103], poverty [95, 102], cognitive impairment [81, 103], presence of physical illness, such as diabetes, heart diseases, stroke and head injury [42, 50, 57, 72, 77, 81, 83, 84, 86–89, 95, 97, 106], living alone [88, 102, 104], disturbed sleep [77, 89], lack of social support [73, 77, 87], dependent totally for the activities of daily living [50, 79, 91, 92, 97, 102, 103], living with family [42, 93], history of a serious life events, such as death in family members, conflict in family, chronic illness in family members and those who had 3 or more serious life events [72, 83, 96], poor daily physical exercise [89] and exposure to verbal and/or physical abuse were strongly and positively associated with depression [90] (Table 2).

**Table 2:** Associated factors for depression among elderly populations

Factor category	Associated factors	AOR	95% CI	Strength of association	Author, year of publication
Demography	> 80 years	NR	NR	NR	Swarnalatha et al. 2013
	Females	NR	NR	NR	
	Illiterates	NR	NR	NR	
Socioeconomic status	Those who were below the poverty line	NR	NR	NR	
	Those who were living alone	NR	NR	NR	
Economic dependency	Those who were economically partially dependent	NR	NR	NR	
ADL	Those depended totally for the activities of daily living	NR	NR	NR	
Sociodemographic characteristics	Female gender	4.75	2.1, 10.7	Strong	Ashe et al. 2019
Socioeconomic status	Low socioeconomic class	9.36	3.69, 23.76	Strong	
Health conditions and comorbidities	Diabetes mellitus	2.76	1.27, 5.98	Moderate	
	Hypertension	2.15	1.06, 4.36	Moderate	
Life events	Death in family members	5.52	2.08, 14.65	Strong	
	Conflicts in family	5.78	2.55, 13.09	Strong	
	Chronic illness in family members	6.77	1.47, 31.13	Strong	
Socio-demographic characteristics	Not married	10.1	3.89, 26.18	Strong	Girma et al. 2016
	Those with no formal education	3.6	1.45, 9.07	Strong	
	Elderly who attended primary school	0.28	0.1, 0.78	Weak	
Substance use and clinical related	Those who had chronic illness	3.47	1.5, 7.7	Strong	
	Elderly with cognitive impairments	2.77	1.18, 6.47	Moderate	
	Substance use	2.6	1.07, 6.28	Moderate	
Socio-demographic characteristics	Female sex	1.72	1.12, 2.66	Weak	Mirkena et al. 2018
	Trading	2.44	1.32, 4.57	Moderate	
	Living with children	3.19	1.14, 8.93	Strong	
	Retirement	3.94	2.11, 7.35	Strong	
Characteristics of the participants	Frequency of children’s visits	NR	NR	NR	He et al. 2016
	Living situation	NR	NR	NR	
	Physical activity	NR	NR	NR	
	Number of chronic diseases	NR	NR	NR	
	Education level	NR	NR	NR	
Demographic characteristics	Lack of social engagement	0.313	0.134, 0.731	Weak	Cong et al. 2015
	Low family support	0.431	0.292, 0.636	Weak	
	Chronic disease	2.378	1.588, 3.561	Moderate	
	Disturbed sleep	1.822	1.187, 2.798	Weak	
Behaviors and life events	Religious belief	3.92	1.18, 13.03	Strong	Feng et al. 2014
	Suffering from more chronic diseases	1.70	1.42, 2.04	Weak	
	Lack of ability to take self-care	2.20	1.09, 4.48	Moderate	
Socio-demographic characteristics	Religion (Non-Christians)	5.67	2.10, 15.27	Strong	Kugbey et al. 2018
	Living arrangement (Alone)	2.36	1.16, 4.83	Moderate	
	Chronic illness (Not having chronic illness)	0.25	0.13, 0.47	Weak	
Socio-demographic and psychosocial profiles	Low income	1.78	1.08, 2.91	Weak	Rajkumar et al. 2009
	Experiencing hunger	2.58	1.56, 4.26	Moderate	
	History of cardiac illnesses	4.75	1.96, 11.52	Strong	
	Transient ischemic attack	2.43	1.17–5.05	Moderate	
	Past head injury	2.70	1.36, 5.36	Moderate	
	Diabetes	2.33	1.15, 4.72	Moderate	
	Having more confidants	0.13	0.06, 0.26	Weak	
Socio-demographic characteristics	Illiteracy	2.01	1.08, 3.75	Moderate	Simkhada et al. 2017
	Physical immobility	5.62	1.76, 17.99	Strong	
	The presence of physical health problems	1.97	1.03, 3.77	Weak	
	Not having any time spent with family members	3.55	1.29, 9.76	Strong	
	Not being considered in family decision-making	4.02	2.01, 8.04	Strong	
Socio-demographic characteristics	Rural habitation	1.6	1.1, 2.4	Weak	Manandhar et al. 2019
	Illiteracy	2.1	1.1, 4.0	Moderate	
Family support	Limited time provided by families	1.8	1.1, 2.9	Weak	
	Exposure to verbal and/or physical abuse	2.6	1.4, 4.8	Moderate	
Sociodemographic–economic characteristics	Female gender	NR	NR	NR	Yaka et al. 2014
	Being single or divorced	NR	NR	NR	
	Lower educational status	NR	NR	NR	
	Low income	NR	NR	NR	
	Unemployment	NR	NR	NR	
	Lack of health insurance	NR	NR	NR	
Baseline characteristics and family relationship	Female sex	2.78	1.54, 7.49	Moderate	Charoensakulchai et al. 2019
	Illiteracy	2.86	1.19, 6.17	Moderate	
	Current smoker	4.25	2.12, 10.18	Strong	
	Imbalanced family type (low attachment, low cooperation and poor alignment between each member)	4.52	2.14, 7.86	Strong	
Sociodemographic characteristics	Not having a main daily activity in men	3.01	1.00, 9.13	Strong	Forlani et al. 2012
Health-Related Variables	Stroke in men	7.25	2.19, 24.06	Strong	
Sociodemographic characteristics	Not living close to friends and family	2.540	1.442, 4.466	Moderate	Wilson et al. 2007
	Poor satisfaction with living accommodation	0.840	0.735, 0.961	Weak	
	Poor satisfaction with finances	0.841	0.735, 0.961	Weak	
	Subsequent development of clinically significant depressive symptoms was associated with base line increased scores in depression	1.68	1.206, 2.341	Weak	
Socio-demographic characteristics	Single elderly	3.27	1.66, 6.44	Strong	Manaf et al. 2016
	Living with family	4.98	2.05, 12.10	Strong	
	Poor general health status	2.28	1.20, 4.36	Moderate	
Clinical characteristics	Heart problems	3.3	1.2, 8.8	Strong	Almeida et al. 2014
ADL	Functional impairment	2.9	2.26, 3.78	Moderate	Weyerer et al. 2008
Socio-demographic characteristics	Smoking	1.6	1.03, 2.36	Weak	
	Multi-domain mild cognitive impairment	2.1	1.30, 3.43	Moderate	
Socio-demographic characteristics	Female gender	10.64	5.09–21.82	Strong	Jadav et al. 2017
	Unemployed/retired	7.37	2.49, 21.79	Strong	
	Illiterate	4.17	1.99, 8.72	Strong	
Clinical related	Respiratory problems	5.47	2.63, 11.37	Strong	
Socio-demographic characteristics	Female sex	NR	NR	NR	Sinha et al. 2013
	Widowhood	NR	NR	NR	
Problems related to social environment	Having no one to talk to (Mild to moderate depression)	3.3	2.5, 4.4	Strong	Kaji et al. 2010
	Having no one to talk to (Severe depression)	5.0	3.6, 6.9	Strong	
Problems with primary support group	Separation/divorce(Mild to moderate depression)	2.8	1.4, 5.3	Moderate	
	Health/illness/care of self(Severe depression)	0.8	0.6, 0.9	Weak	
Socioeconomic characteristics	Socioeconomic deprivation at municipal levels	1.16	1.04, 1.30	Weak	Ferna´ndez et al. 2014
Socio-demographic characteristics	Poor education	NR	NR	NR	Al-Shammari et al. 1999
	Unemployment	NR	NR	NR	
	Divorced or widowed status	NR	NR	NR	
	Old age	NR	NR	NR	
	Being a female	NR	NR	NR	
	Living in a remote rural area with poor housing arrangements	NR	NR	NR	
	Limited accessibility within the house and poor interior conditions	NR	NR	NR	
	Limited privacy, such as having a particular room specified for the elderly	NR	NR	NR	
	Lower incomes inadequate for personal needs as well as depending on charity or other relatives	NR	NR	NR	
Socio demographic Profile	Unemployment	NR	NR	NR	Sidik et al. 2004
Socio-demographic Status	Aged 75 to 84 years	2.1	1.1, 3.9	Moderate	Subramaniam et al. 2016
	Those of Indian ethnicity	4.1	1.1, 14.9	Strong	
	Those of Malay ethnicity	5.2	3.1, 8.7	Strong	
Other Health Conditions	Those who had a history of depression diagnosis by a doctor	3.2	1.9, 5.4	Strong	
Socio-demographic characteristics	Being retired	3.88	1.27, 11.76	Strong	Assil et al. 2013
	Having social problems	3.27	1.45, 7.41	Strong	
	Having living problems	2.19	1.19, 3.94	Moderate	
Physical illness	Those who had 4 or more infirmity	2.08	NR	Moderate	Haseen et al. 2011
Disability Assessment	Those who had medium disability	3.12	NR	Strong	
Serious life events	Those who had 3 or more serious life events	5.25	NR	Strong	
Socio-demographic characteristics	Female gender	1.8	NR	Weak	Ghubash et al. 2004
	Insufficient income	3.8	NR	Strong	
	Being single, separated, divorced or widowed	2.1	NR	Moderate	
Socio-demographic Characteristics	Age ≥ 75 years	5.08	2.21, 11.89	Strong	Abdo et al. 2011
	Being female	2.56	1.55, 4.24	Moderate	
	Not married	4.47	2.52, 7.97	Strong	
	Having previous death event among the surrounding	7.68	3.57, 16.93	Strong	
Respondent characteristics	Years of education	0.87	NR	Weak	McCall et al. 2002
	Difficulties performing activities of daily living	1.72	NR	Weak	
	Enrolled in medicaid	2.67	NR	Moderate	
Socio-demographic variables	Being female Residing in rural or suburb	1.25 2.31	1.02, 1.54 1.88, 2.86	Weak Moderate	Li et al. 2016
	Currently not married or not living with spouse	1.45	1.17, 1.80	Weak	
	Poor physical health	5.23	3.97, 6.88	Strong	
	Poor daily physical exercise	1.79	1.39, 2.29	Weak	
	Poor sleep quality	2.76	2.14, 3.56	Moderate	
Socio-demographic variables	Low educational level	5.9	1.5, 22.6	Strong	Mendes-Chiloff et al. 2008
	Death	5.5	1.7, 17.1	Strong	
ADL	Dependence regarding basic ADL	5.1	2.2, 11.0	Strong	
Socio-demographic variables	Illiterate or elementary school	1.68	1.2, 2.29	Weak	Li et al. 2016
	Poor physical health	4.49	(3.15, 6.38	Strong	
	Poor daily physical exercise	1.51	1.07, 2.11	Weak	
	Poor sleep quality	3.25	2.33, 4.53	Strong	
Socio-demographic	Financial fears regarding future	NR	NR	NR	Prashanth et al. 2015
	Income insufficiency	NR	NR	NR	

AOR: Adjusted Odds Ratio; CI: Confidence Interval; NR: Not Reported

## Discussion

As to the researcher’s knowledge, this review and meta-analysis on the prevalence and determinants of depression among old age are the first of their kind in the world. Therefore, the knowledge generated from this meta-analysis on the pooled prevalence and associated factors for depression among old age could be important evidence to different stakeholders aiming to plan policy in the area. The average prevalence of depression among old age using the random effect model was found to be 31.74%. A subgroup analysis was done considering the economic status of countries, the study instrument, and the sample size of each study.

In the present systematic review and meta-analysis, the existing available information varies by the region, where the study was conducted, data collection tools used to screen depression, and the sample size assimilated in the study. Sixty-two percent (*n* = 26) of the studies were found in developing countries. About 38% (*n* = 16) of the incorporated studies utilized GDS-15 to screen depression, around 28% (*n* = 12) studies used GDS-30 to screen depression, ten percent (*n* = 4) studies used GMS to screen depression, whereas the rest utilized other tools. More than half (*n* = 22) of the included studies utilized a sample size of below 450.

The result of this meta-analysis revealed that depression in the elderly populations in the world was high (31.74%). This pooled prevalence of depression among old age in the world (31.74%; 95% CI 27.90 to 35.59%) was higher than a global systematic review and meta-analysis study on 95,073 elderly populations aged > 75 years and 24 articles in which a pooled prevalence of depression was 17.1% (95% CI 9.7 to 26.1%) [107], a global systematic review and meta-analysis study on 41 344 outpatients and 83 articles in which a pooled prevalence of depression was 27.0% (95% CI: 24.0% to 29.0%) [108], WHO reports on mental health of older adults over 60 years old with 7% prevalence of depression in the general older population [106], a Brazilian systematic review and meta-analysis study on 15,491 community-dwelling elderly people average age 66.5 to 84.0 years and 17 articles with a pooled prevalence rates of 7.0% for major depression, 26.0% for CSDS (clinically significant depressive symptoms), and 3.3% for dysthymia [109] and an Iranian meta-analysis study on 3948 individuals aged 50 to 90 years and 13 articles with a pooled prevalence of severe depression was 8.2% (95% CI 4.14 to 6.3%) [110]. The reason for such a high prevalence of depression in the globe would be due to the difference in sample size, study subjects, the severity of depression, study area, study instruments, and the means of administration of the tools employed in the studies [111].

In contrast to our current systematic review and meta-analysis study, the pooled prevalence of depression was lower than a Chinese Meta-Analysis of Observational Studies on 36,791 subjects and 46 articles with a pooled prevalence of depression was 38.6% (95% CI 31.5–46.3%) [112], and an Indian systematic review and meta-analysis study on 22,005 study subjects aged 60 years and above, and 51 articles with a pooled prevalence of depression was 34.4% (95% CI 29.3 to 39.6) [113]. The reason for the discrepancy might be due to the wide coverage of the study and the higher sample size utilized in the present study. Furthermore, differences could be due to the poor health care coverage and significant population makes a destitute life both in China and India. In addition, both China and India have a rapidly aging population. Old age causes enforced retirement which may lead to marginalizing older people. Elders are regarded as incompetent and less valuable by potential employers. This attitude serves as a social stratification between the young and old and can prevent older men and women from fully participating in social, political, economic, cultural, spiritual, civic, and other activities [114–116].

A significant regional variation on the pooled prevalence of depression in the elder population was observed in this review and meta-analysis study. The aggregate prevalence of depression in elderly population among developing countries; 40.78% [38, 42, 69–73, 75, 76, 78, 81–83, 86, 88, 90, 92–98, 101, 102, 105] was higher than the prevalence in developed countries; 17.05% [50, 57, 74, 77, 79, 80, 84, 85, 87, 89, 91, 99, 100, 103, 104]. The huge variation might be due to absolute poverty, economic reform programs, limited public health services, civil unrest, and sex inequality are very common in developing countries [117].

Likewise, the greater pooled prevalence of depression in elderly population was observed in studies using a sample size below 450 study subjects (40.12%) [38, 42, 57, 70–73, 75, 76, 79, 81, 86, 88, 90, 92, 94, 96–99, 102, 104] than the pooled prevalence of depression in elders that used a sample size of 450–999 (25.38%) [74, 80, 82, 84, 85, 91, 93, 95, 100, 105], and above 1000 (20.19%) [50, 69, 74, 77, 78, 83, 87, 89, 101, 103]. The reason could be a smaller sample size increases the probability of a standard error thus providing a less precise and reliable result with weak power.

Regarding the associated factors; being female, age older than 75 years, being single, divorced or widowed, being unemployed, retired, no educational background, low level of education, low level of income, lack of social support, living with family, current smoker, presence of physical illness, such as diabetes, heart diseases, stroke, and head injury, poor sleep quality, physical immobility and a history of serious life events, such as a death in family members, conflict in the family, chronic illness in family members and those who had 3 or more serious life events were found to have a strong and positive association with depression among old age.

### Difference between included studies in the meta-analysis

This meta-analysis study was obtained to have a high degree of heterogeneity between the studies incorporated in pooling the prevalence of depression in the elderly population of the world. The analysis of subgroups for detection of sources of heterogeneity was done and the economic status of the country, where the study was done, data collection instruments, and sample size were identified to contribute to the existing variation between the studies incorporated in the analysis. Besides, a sensitivity analysis was performed using the random-effects model to identify the effect of individual studies on the pooled estimate. No significant changes in the pooled prevalence were found on the removal of a single study.

Limitations should be considered when interpreting the results of this study. Screening tools cannot take the place of a comprehensive clinical interview for confirmatory diagnosis of depression. Nevertheless, it is a useful tool for public health programs. Screening provides optimum results when linked with confirmation by mental health experts, treatment, and follow-up. As this meta-analysis included studies done using screening tools, a further meta-analysis done with diagnostic tools will help to assess the true burden of depression and to determine the need for pharmacological and non-pharmacological interventions. Furthermore, because of the lack of access to the full text of some studies, the researchers failed to include these research findings.

## Conclusion

This review and meta-analysis study obtained a pooled prevalence of depression in the elderly population in the world to be very high, 31.74% (95% CI 27.90, 35.59). This pooled effect size of depression in the elderly population in the world obtained is very important as it showed aggregated evidence of the burden of depression in the targeted population. Since the high prevalence of depression among the old population in the world, this study can be considered as an early warning and advice to health professionals, health policymakers, and other pertinent stakeholders to take effective control measures and periodic assessment for the elderly population.

## Data Availability

The data sets used and/or analyzed during the current study are available from the corresponding author on reasonable request.

## Abbreviations

ADL: Activities of daily living
AOR: Adjusted odds ratio
CDEP: Community-dwelling elderly people
CES-D: Center for Epidemiologic Studies Depression Scale
CI: Confidence interval
CIDI-SF: Composite International Diagnostic Interview Short Form
CSDS: Clinically significant depressive symptoms
CS: Cross-sectional
DASS-21: Depression, Anxiety, and Stress Scale
DSM-III: Diagnostic and Statistical Manual of Mental Disorders
EMI: Elderly medical inpatients
GD: Geriatrics depression
GDS: Geriatric Depression Scale
GMS: Geriatric Mental State Schedule
HADS: Hospital Anxiety and Depression Scale
KICA-dep: Kimberley Indigenous Cognitive Assessment of Depression
MCS: Mental Component Summary
NR: Not reported
PRISMA: Preferred Reporting Items for Systematic Reviews and Meta-analysis
UK: United Kingdom
USA: United States of America
WHO: World Health Organization
